# Employing Synergetic Effect of Doping and Thin Film Coating to Boost the Performance of Lithium-Ion Battery Cathode Particles

**DOI:** 10.1038/srep25293

**Published:** 2016-05-04

**Authors:** Rajankumar L. Patel, Ying-Bing Jiang, Amitava Choudhury, Xinhua Liang

**Affiliations:** 1 Department of Chemical and Biochemical Engineering, Missouri University of Science and Technology, Rolla, Missouri 65409, United States; 2 TEM Laboratory, University of New Mexico, Albuquerque, New Mexico 87131, United States; 3 Department of Chemistry, Missouri University of Science and Technology, Rolla, Missouri 65409, United States

## Abstract

Atomic layer deposition (ALD) has evolved as an important technique to coat conformal protective thin films on cathode and anode particles of lithium ion batteries to enhance their electrochemical performance. Coating a conformal, conductive and optimal ultrathin film on cathode particles has significantly increased the capacity retention and cycle life as demonstrated in our previous work. In this work, we have unearthed the synergetic effect of electrochemically active iron oxide films coating and partial doping of iron on LiMn_1.5_Ni_0.5_O_4_ (LMNO) particles. The ionic Fe penetrates into the lattice structure of LMNO during the ALD process. After the structural defects were saturated, the iron started participating in formation of ultrathin oxide films on LMNO particle surface. Owing to the conductive nature of iron oxide films, with an optimal film thickness of ~0.6 nm, the initial capacity improved by ~25% at room temperature and by ~26% at an elevated temperature of 55 °C at a 1C cycling rate. The synergy of doping of LMNO with iron combined with the conductive and protective nature of the optimal iron oxide film led to a high capacity retention (~93% at room temperature and ~91% at 55 °C) even after 1,000 cycles at a 1C cycling rate.

LiMn_1.5_Ni_0.5_O_4_ (LMNO) has received much attention as alternate cathode materials for lithium ion batteries (LIBs) due to its improved cycling behavior relative to the pristine spinel^1^. The operating voltage window of LMNO makes it a potential candidate for use in hybrid electric vehicles (HEV) due to its nominal cost, enhanced thermal stability and enhanced rate capability owing to its three-dimensional structure^2^,^3^,^4^,^5^. However, it has not gained commercial usability in HEV due to high capacity fade during cycling at elevated temperatures and Mn^3+^ dissolution by HF^6^,^7^. Doping LMNO with ions has been considered to be an effective way to better the core properties of LMNO for enhanced electrochemical performance. In a typical cycling curve of LiNi_0.5− x_Fe_x_Mn_1.5_O_4_, the oxidation-reduction pairs, Fe^IV^/Fe^III^, Ni^IV^/Ni^II^, and Mn^IV^/Mn^III^, correspond to the three stages of addition or subtraction of electrons in and out of the d-spacing of the compounds^8^. Liu *et al.*^9^ reported that Fe doping could subdue the solid electrolyte interface (SEI) formation by enabling certain surface enhancements, which is very important to improve the electrochemical performances of the 5 V spinel cathode materials. Doping alone cannot, however, significantly improve the cycleability and capacity retention of LMNO as it cannot avoid dissolution of Mn^3+^ ions by HF^10^. One approach to solve this problem is to form a protective film around the LMNO structure, thereby significantly reducing Mn^3+^ dissolution and improving capacity retention and cycling performance.

Several researchers have used wet chemical methods including sol-gel method to coat protective film over pristine LMNO^11^,^12^. The protective coating improved cycling life and capacity retention of LMNO. However, there was always a bargain between increasing the capacity and longer cycle life of the battery. In these studies, it was difficult to precisely control the thickness of the coating, and the films were not conformally coated on the particle surfaces. The increased thickness causes increased mass transfer resistance that delays the movement of species, electrons, and ions. Hence, an optimal thickness of the film is crucial for the best performance enhancement. This can be achieved by atomic layer deposition (ALD).

ALD is best known for its ability to deposit high-quality, ultra thin conformal films of materials based on alternating dosing of chemical vapors that react with surfaces^13^,^14^,^15^,^16^. ALD ensures control of film thickness at nanometer level. However, if the ALD film is insulating, such trade-off still exists^14^, which has also been demonstrated in our recent work and an effective solution was proposed^17^. It has been demonstrated that coating an optimal thickness of conductive metal oxide films could improve both specific capacity and cycling performance. In our recent work^17^, ultrathin 3 nm ALD CeO_2_ film was coated on LiMn_2_O_4_ particles that showed 24% improvement in specific capacity, compared to the uncoated one, and high capacity retention of 96% at room temperature and 95% at an elevated temperature of 55 °C even after 1,000 cycles at a 1C rate. Herein, we propose that iron oxide would be an excellent candidate as the coating layer due to its electrochemical activity, low cost, environmental benignity, and natural abundance. The conductive nature of iron oxide coating using sputtering or liquid phase method has improved electrochemical performance of carbon nanotubes^18^ and SnO_2_ particles^19^, but the results were restricted to small number of cycles and the coatings were not optimal to make it a viable solution. Conformal iron oxide films with controlled thickness can be coated by ALD. Due to the relative high temperature of iron oxide ALD coating process and the surface defects of LMNO, we expected that Fe could also be doped in the crystal strucutre of LMNO.

To the best of our knowledge, there has been no successful study, so far, exploring the synergetic effect of iron doping and ultra thin film coating of iron oxide using ALD on LIB electrode particles. In this study, large quantities of LMNO particles were coated with ultra thin iron oxide films by ALD in a fluidized bed reactor. The ALD coated samples demonstrated both longer cycle life with improved stable performance for more than 1,000 cycles of electrochemical cycling at room temperature and at 55 °C. We also report the first time a unique phenomenon of ionic Fe entering the lattice structure of LMNO during the ALD coating process. We believe the combined effect of the doping of Fe into the structure of LMNO and the conductive optimal ultrathin coating of iron oxide films has significantly enhanced cycleability and reduced capacity fade of LMNO.

## Results and Discussion

### Iron oxide films coated on LiMn_1.5_Ni_0.5_O_4_ particles

**Figure 1:**
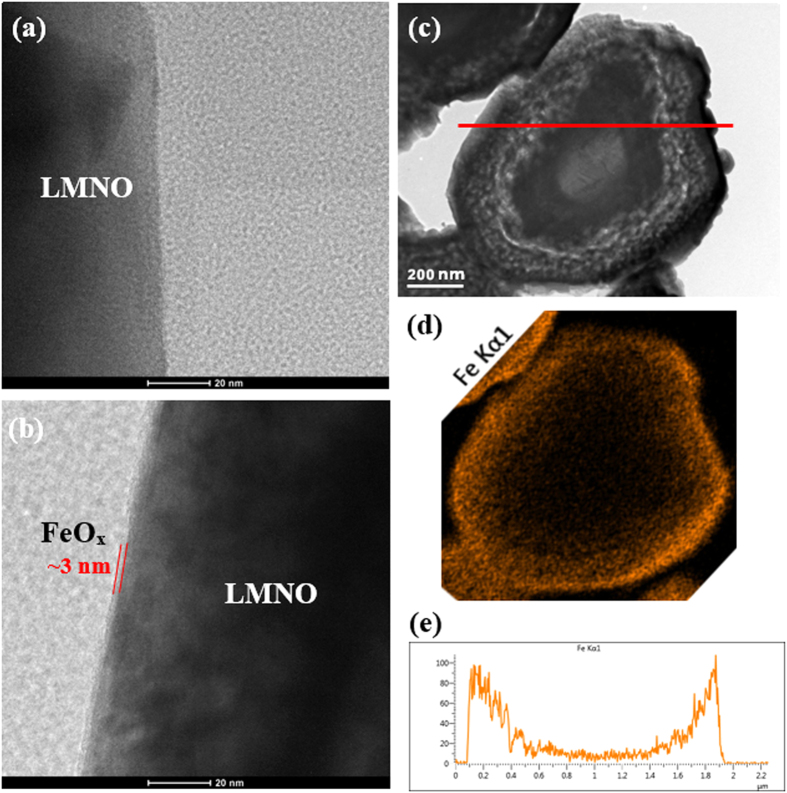
TEM images of (**a**) clean edge of an uncoated LiMn_1.5_Ni_0.5_O_4_ particle, and (**b**) ~3 nm of conformal iron oxide film coated on one LiMn_1.5_Ni_0.5_O_4_ particle after 160 cycles of iron oxide ALD, (**c**) cross sectional TEM image of one LiMn_1.5_Ni_0.5_O_4_ particle with 160 cycles of iron oxide ALD, (**d**) Fe element mapping of cross-sectioned surface by EDS, and (**e**) Fe EDS line scanning along the red line as shown in (**c**). TEM image indicates that conformal iron oxide films were coated on primary LiMn_1.5_Ni_0.5_O_4_ particle surface. EDS mapping and EDS element line scanning indicates that Fe was doped in the lattice structure of LiMn_1.5_Ni_0.5_O_4_.

Different numbers of iron oxide ALD coating cycles were applied on the surfaces of LMNO particles (4–5 μm, NANOMYTE^®^ SP-10, NEI Corporation). ALD reaction was carried out for 10 (10Fe), 20 (20Fe), 25 (25Fe), 30 (30Fe), 40 (40Fe), 80 (80Fe), and 160 (160Fe) cycles. The transmission electron microscopy (TEM) image of an uncoated (UC) LMNO particle (shown in Fig. 1a) displays a blank edge of a pristine particle. In contrast, a distinctive conformal coating of ~3 nm layer on a LMNO particle after 160 cycles of iron oxide ALD, is seen in Fig. 1b. Figure S1a –d (see supporting information) show images at different magnification level for one particle. It is clear from the series of images that the iron oxide coating was conformal and covering the entire particle surface. Based on this 160Fe sample, the growth rate of iron oxide films on the LMNO particles was ~0.02 nm/cycle. The iron oxide growth rate is in sync with the previously reported values^20^. The growth rate value is derived from TEM images only and it does not represent the actual number of layers since ALD process experiences nucleation period at the beginning of the cycles. The Fig. S2a,b (see supporting information) show the selective area electron diffraction (SAED) pattern of those two samples. Both powders exhibited well-developed octahedral shapes, although a secondary phase appeared to grow on the corner of the octahedral particle after coating 160 cycles of iron oxide ALD, as indicated in Fig. S2b. In order to confirm the diffusion and distribution of iron inside the particle structure, about 80 nm thick thin section across the center of the 160Fe sample particle was cut using focused-ion beam (FIB) and elemental mapping was performed using energy dispersive x-ray spectroscopy (EDS). Figure 1c is the regular TEM image of the thin-section across the center of a particle. Figure 1d is the Fe elemental mapping of the same particle as shown in Fig. 1c, acquired in the scanning TEM (STEM) mode combined with EDS collection. Figure 1e is the Fe element distribution along the red line as shown in Fig. 1c, and EDS line scan in the STEM mode was used to acquire this information. It clearly shows the Fe penetration ~400 nm deep below the 160Fe LMNO particle surface. The EDS spectrum from the surface vicinity of the UC and the 160Fe samples are shown in Fig. S3. It is evident from those spectra that there was no Fe in the UC sample, while there was a large amount of Fe on the particle surface of the 160Fe sample. This study in addition to the TEM images (as in Fig. 1b) provides evidence needed to support the claim that the doping and coating both occurred during the ALD coating process. This unique phenomenon has never been reported during ALD coating process.

The Fe content on the LMNO particles was measured using inductively coupled plasma atomic emission spectroscopy (ICP-AES). As shown in Fig. S4 in supporting information, iron content increased almost linearly with increase in the number of ALD cycles. The thicknesses of iron oxide films were reflected by the content of Fe on the particles. The iron oxide film thickness was several magnitudes smaller than the 4–5 micron sized cathode particles. The plot trend clearly indicated a linear growth rate of iron oxide ALD films onto the particles surface except for a short initial period for the first 10 ALD cycles. The surface area of the UC samples was 1.8 m^2^/g measured by using Quantachrome Autosorb-1. Based on the surface area of particles, percentage of Fe in the 160Fe sample obtained from ICP-AES, and assuming the oxide films being Fe_3_O_4_, the expected thickness of the ultrathin film was found to be ~10 nm. However, the TEM analysis showed the film thickness to be only 3 nm. This discrepancy also indirectly supported that Fe had entered the lattice structure of LMNO.

**Figure 2:**
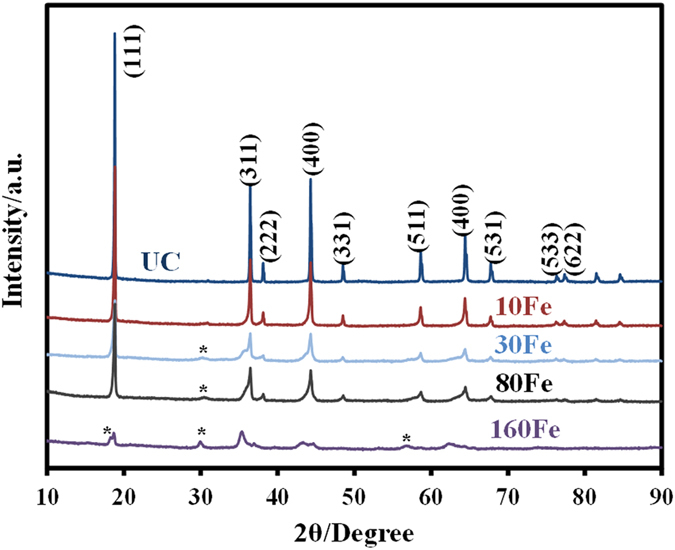
XRD patterns of uncoated LiMn_1.5_Ni_0.5_O_4_ particles and coated with different cycles of ALD iron oxide. * indicates the dominant Fe_3_O_4_ phase due to iron oxide ALD coating.

Figure 2 shows the powder X-ray diffraction (PXRD) pattern of the UC, 10Fe, 30Fe, 80Fe, and 160Fe samples. The PXRD patterns of pristine and modified samples confirm the existence of cubic spinel structure. All the main diffraction peaks are sharp, which indicates that the tested samples are well-crystallized. The pattern for the UC differs significantly from the 160Fe sample. For the 160Fe sample, the main peaks are not so sharp and some of the peaks have a significant shift in their position, indicating the significant amount of Fe substituted into the LMNO structure. The weak reflections observed at around 18.2°, 30°, and 57.5° in the 160Fe sample are absent in the 10Fe sample and only 30° peak in 30Fe and 80Fe. The presence of Fe_3_O_4_ was confirmed for the case of 160Fe by the additional peaks at 30° and 57.5°, which are consistent with the reported results^21^,^22^,^23^. The PXRD patterns, consistent with the SAED pattern, indicate that the iron oxide ALD coated LMNO does not have the same phase as its uncoated counterpart. X-ray photoelectron spectroscopy (XPS) results further confirmed the presence of Fe_3_O_4_ phase in the 160Fe sample (see Fig. S5 in supporting information). For the 30Fe and 40Fe samples, the Fe content was much lower than that of the 160Fe sample, and PXRD showed very weak peaks to indicate the presence of Fe. Iron content in 10Fe was too low to detect any particular iron oxide phase confidently. This all could be explained with the fact that the ALD deposition of iron oxide using ferrocene and oxygen precursors at high temperature (in this case 450 °C) resulted in formation of Fe_3_O_4_ as evidence from the PXRD^24^,^25^, which could be pure Fe_3_O_4_ spinel with Fe_tet_^3+^[Fe^2+^Fe^3+^]_oct_O_4_ (magnetite) composition, a defect non-stoichiometric spinel, Fe_3−x_O_4_ or γ-Fe_2_O_3_ (maghemite). γ-Fe_2_O_3_ is the end member of non-stoichiometric Fe_3−x_O_4_, given as Fe_tet_^3+^[Fe_5/3_^3+^□_1/3_]_oct_O_4_ [□ represents vacant site]^26^. Unfortunately, PXRD of these phases have subtle differences, which make it difficult to distinguish between them especially when the amount of Fe-content is less and particle sizes are small. To get a better insight into the nature of Fe_3_O_4_ phase, we carried out Mössbauer spectroscopy of the 160Fe sample, since this sample had substantial amount of Fe for reliable Mössbauer signal. The room temperature (25 °C) Mössbauer spectrum of 160Fe exhibits a broad sextet indicating the presence of hyperfine magnetic component together with a central quadrupolar doublet (Fig. S6 in supporting information). The broadness of resonance lines in sextet is an indication of small particle size and a distribution of hyperfine magnetic fields. The isomer shift (δ), quadrupolar splittings (Q.S.) and hyperfine field (B_hf_) of the sextet are 0.32(5) mm/s, 0.016(6) mm/s, and 44.6(5)T, respectively, consistent with γ-Fe_2_O_3_ and rules out possibility of octahedral Fe^2+^ as in spinel Fe_3_O_4_, which produces another sextet subspectra with high δ value (~0.63 mm/s)^27^. The δ and Q.S. for the quadrupolar splitting for the central doublet are 0.36 and 0.74 mm/s, respectively. The δ and Q.S. values for the central doublet are characteristic of Fe^3+^ ions in octahedral coordination^28^, which may arise from the doping of Fe^3+^ in LMNO phase as hypothesized based on the TEM studies and shifting of PXRD lines of coated LMNO with respect to pristine sample. However, there is a note of caution here; such central doublet can also arise due to the presence of superparamagnetic iron-oxide particles. In summary, the results from PXRD, TEM-SAED, STEM-EDS, and XPS strongly suggest that during the ALD coating process, some amount of Fe doping occurred (in some valance state Fe penetrated into the lattice structure of LMNO) and Fe_3_O_4_ ultra thin film formed. With increment in iron oxide ALD cycles, Fe_3_O_4_ can be further oxidized to provide γ-Fe_2_O_3_^29^, as here in the case of 160Fe.

### Electrochemical testing

**Figure 3:**
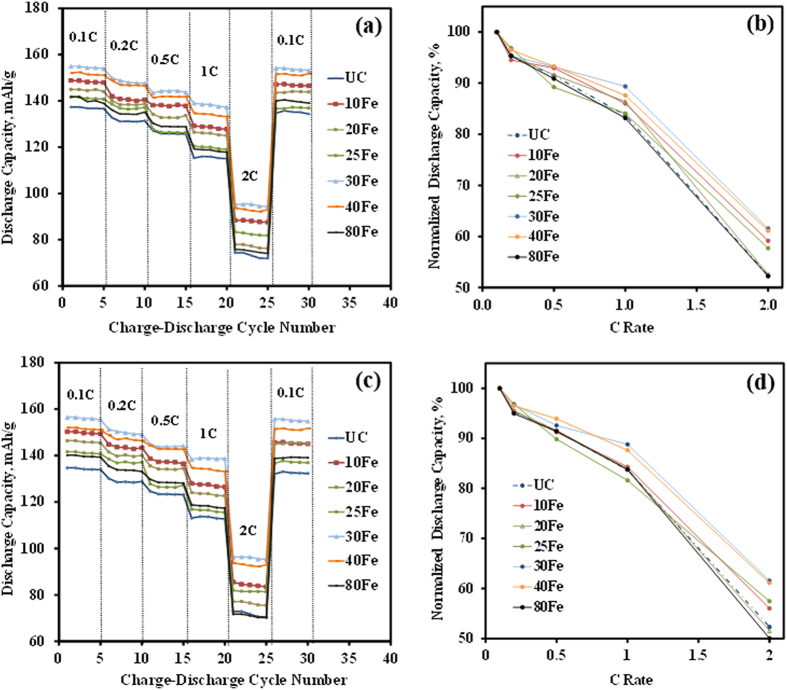
Galvanostatic discharge capacities of cells made of LiMn_1.5_Ni_0.5_O_4_ particles coated with different thicknesses of iron oxide at different C rates in a voltage range between 3.5–5 V (**a**) at room temperature and (**c**) at 55 °C; in (**b**,**d**) their respective normalized capacity vs. rate curves.

The charge-discharge analysis was carried out in a 3.5 V–5 V voltage range. Figure 3a,c show the discharge capacities of the UC, 10Fe, 20Fe, 25Fe, 30Fe, 40Fe and 80Fe samples that were discharged at different C rates, of 0.1C, 0.2C, 0.5C, 1C, and 2C, for five cycles at room temperature and 55 °C, respectively. For these conditions, almost all of the iron oxide ALD coated samples showed higher initial discharge capacity than the UC. The increased discharge capacity of iron oxide coated samples can be attributed to synergetic effect between doped Fe and conductive iron oxide overlayer. In Fig. 3b, the normalized discharge capacities obtained at various C rates are plotted for all samples in reference to capacity obtained at 0.1 C. The results clearly demonstrate that the 30Fe sample showed superior rate capability as compared to other samples at room temperature. At a 2C rate where charge-discharge cycle was about 30 min, the 80Fe sample performed poor due to the increased mass transfer resistance caused by the thicker coating. At 55 °C, in Fig. 3d, a similar trend is observed. Overall the 30Fe sample performed better than any other coated or uncoated samples. At a 2C rate, the 80 Fe sample performed much poorer as compared to room temperature testing due to degradation of cell performance at high temperature. The diffusional and kinetic overpotential, solid electrolyte interphase (SEI) layer induced resistance, and contact/ohmic resistance are the main causes of the voltage drop in a typical LIB. The ultrathin iron oxide ALD film can significantly alter the most of these causes of the voltage drops. However, if the Li concentration ratio between the particle surface and the bulk is not affected by the coating, then the overpotential caused by the diffusional forces remains unchanged. The layer formed on the cathode surface (known as solid permeable interface) is usually much thinner than the SEI layer formed on the anode surface, and its thickness increases with charge-discharge cycling and the temperature^30^.

**Figure 4:**
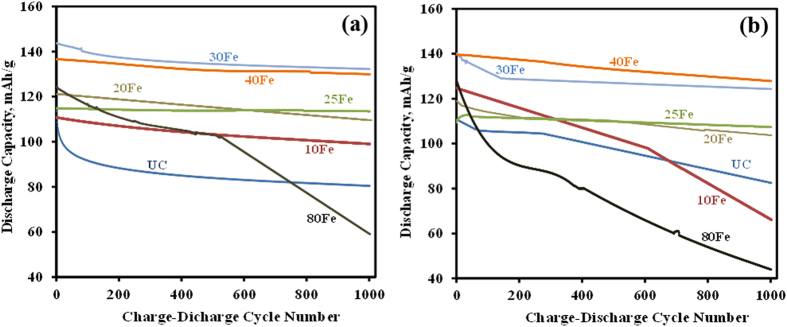
Discharge capacities of cells made of LiMn_1.5_Ni_0.5_O_4_ particles coated with different thicknesses of iron oxide at a 1C rate in a voltage range between 3.5–5 V (**a**) at room temperature and (**b**) at 55 °C.

Figure 4a shows the results of discharge cycling at a 1C rate between 3.5 V–5 V for the UC, 10Fe, 20Fe, 25Fe, 30Fe, 40Fe, and 80Fe cells at room temperature up to 1,000 cycles. The discharge capacity of the UC was initially 114 mAh/g, and it declined to 80 mAh/g after 1,000 cycles. In contrast, the 30Fe and 40Fe samples exhibited much higher initial discharge capacities than the UC. The 30Fe showed a remarkable initial discharge capacity of 143 mAh/g, which is ~25% increment compared to the UC. The differance between 30Fe and 40Fe became much less with increase in cycle numbers. The stable discharge capacity at ~133 mAh/g was maintained (which is ~19 mAh/g higher than the UC cell’s initial capacity) for the case of 30Fe even after 1,000 cycles, which means that it dropped only by less than 7%, compared to its initial capacity. Similarly, 40Fe showed remarkable ~95% capacity retention after 1,000 cycles at room temperature. This is the only time when 40Fe showed better results than 30Fe. The reason is not apparent, but it could be argued that the structural similarity of the iron oxide film and perhaps the amount of doped Fe are the reason that 30Fe and 40Fe showed very comparable results throughout this study. In addition, as seen in Fig. 4b, the ALD coated LMNO showed significantly improved cycling performance, even at an increased testing temperature of 55 °C. The 30Fe and 40Fe cells exhibited an initial discharge capacity of ~140 mAh/g. After 1,000 cycles, the capacity of 30Fe was stabilized at around 125 mAh/g after a gradual decrease from its initial capacity. The 30Fe and 40Fe samples showed much higher capacity than the UC sample, which indicated that iron oxide coated LMNO particles was much more chemically and thermally stable. The 10Fe sample showed higher initial capacity than the 20Fe and 25Fe, which is in sync with the different C rate results. However, in a long run, it declined very significantly. This could be explained by the same reason that the Fe doped into the structure of the LMNO helped improve the initial capacity of the material and the iron oxide coating which occured after more ALD cycles (as in 20Fe and 25Fe) gave stability to the material. The 80Fe sample showed poor stability over the testing time of 1,000 charge-discharge cycles. The reason could be that it has relatively thicker coating than other coated samples. The thick film induces more stresses during lithium ion insertion and deinsertion. These increased stresses combined with more mass transfer resistance of Li^+^ due to the relatively thick films as compared to 30Fe/40Fe lead to poorer performance of the 80Fe sample. With increase in charge-discharge cycling, less Li^+^ inserted into cathode due to the increasing thickness of the SEI layer on lithium. This explains the worst performance of the 80Fe sample.

**Figure 5:**
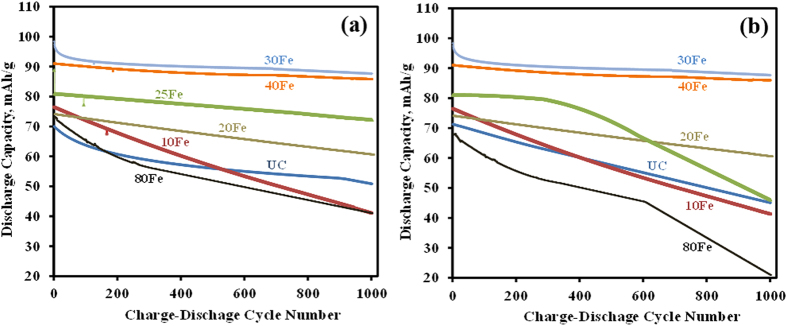
Discharge capacities of cells made of LiMn_1.5_Ni_0.5_O_4_ particles coated with different thicknesses of iron oxide at a 2C rate in a voltage range between 3.5–5 V (**a**) at room temperature and (**b**) at 55 °C.

As learned from our previous work^17^, the drawback of coating on particles is slower species transport. Consequently, a demonstration of performance improvement via ALD coatings at a high C rate is significant because the diffusivity of ions in the solid phase becomes significant as the input current increases. Also, the inside temperature of a cell increases with faster charge-discharge cycle rate, and that also increases the stress level due to developed concentration gradient inside particles. There is also a possibility of phase transition at the particle surface from overlithiation during this cycling process. So, in order to examine the perfomance of these coated samples, they were cycled at a 2C rate, shown in Fig. 5. The performance of 10Fe improved slightly due to initial iron doping. The trend is similar to the test at a 1C rate as discussed earlier and the higher initial capacity of 10Fe did not last longer than the 20Fe and 25Fe coated samples. A conformal coating of iron oxide with a larger number of ALD coating cycles provided a protection, which resulted in a significant improvement in initial capacity fade and remarkable stable performance, as in the case of 30Fe. The 30Fe and 40Fe samples still had far better discharge capacity and stability than the UC sample, even after 1,000 cycles at a 2C rate at both room temperature and 55 °C. The 30Fe and 40Fe samples showed more than 90% capacity retention after 1,000 cycles, while the UC sample could not withstand the high rate of charge-discharge cycling and the capacity kept dropping. Overall, the 30Fe cells showed consistently better than any other prepared cells. The excellent cycling behavior of the iron oxide ALD-coated LMNO electrodes, compared to the UC cell, clearly indicates that the synergetic effect of ALD deposited iron oxide coating and Fe doping into the LMNO structure (see STEM-EDS and TEM-FIB results) could well be the reason for the significantly improved electrochemical performances even at high C rates and high temperature cycling.

**Figure 6:**
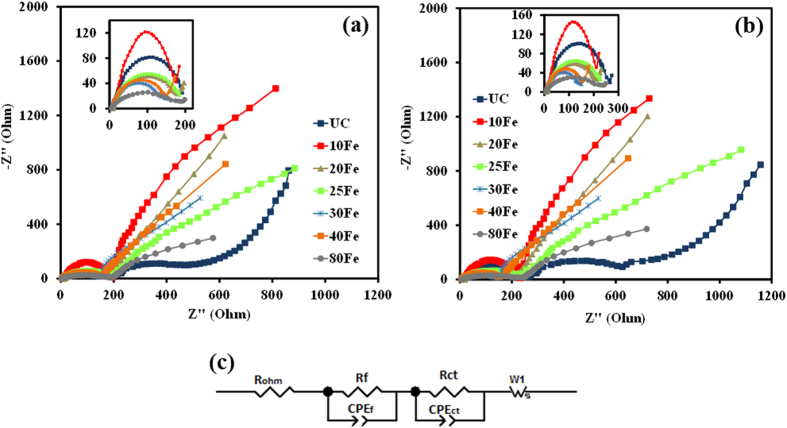
Electrochemical impedance spectra at room temperature for uncoated LiMn_1.5_Ni_0.5_O_4_ particles and coated with various thicknesses of iron oxide after (**a**) 1^st^ cycle and (**b**) 1,000^th^ charge-discharge cycles, and (**c**) equivalent circuit fit for the impedance spectra. Inset images show the high frequency regions (1M Hz–100 Hz) of the impedance spectra.

The interface change due to ALD thin film coating was further investigated using electrochemical impedance spectroscopy (EIS). A three electrode configuration was used for the EIS measurements. The cathode in the coin cell served as the working electrode, whereas Li metal anode served as both the reference and the counter electrode. All the impedance measurements were performed at open circuit voltage. The impedance spectra were fitted using equivalent circuit that consisted of three resistance elements, two constant phase elements and a warburg diffusion element (see Fig. 6c). The details about the fitted parameters are explained elsewhere^17^. Among the fitted parameters, ohmic resistance (R_ohm_), charge-transfer resistance (R_ct_), and surface film resistance (R_f_) can be used to quantify the polarization behaviors. The W1 element represents the warburg impedance, which can be used to quantify Li-ion mass transfer resistance. Tables 1 and 2 provide the list of all the fitted parameters value obtained after fitting the impedance curves to an equivalent circuit. The semicircle from the impedance analysis of all the cells was fitted using a combination of two R/C units (resistor/capacitor) to represent surface-film and charge-transfer resistance, R_(f+ct)_. For clarification, the lines in resultant impedance curves were not obtained after fitting the equivalent circuit to the impedance curves. One semicircle was observed for the UC and the iron oxide ALD coated cells, as shown in Fig. 6. Upon a close look at the semicircle, it reveals that they in fact are two semicircles overlapped, which could be contributed from the SEI film (at higher frequency region) and the charge-transfer resistance at the particle surface (at mid to high frequency regions)^9^,^14^. After the 1^st^ and 1,000^th^ charge-discharge cycles, the radius of the semicircles of the 30Fe and 40Fe cells are smaller in comparison to the UC cell, as evident in Fig. 6a,b. With the increase in the thickness of iron oxide ALD films, the radius of the semicircle increased, as in the case of 80Fe, which was mainly due to the increased charge-transfer resistance (see Tables 1 and 2), indicating that the sluggish transit of Li through the longer pathway. After 1,000 charge-discharge cycles, the warburg resistance (the element that is representative of Li^+^ ion diffusion resistance) was the highest for the UC sample as compared to the coated samples. The charge transfer resistance first decreased with increase in the number of ALD coating cycles, reached a minimal value for the 30Fe sample and then increased with the increase in number of ALD coating cycles. This trend is indicative that 30Fe sample has the optimal thick coating as comapred to the other samples. The film resistance also followed a similar trend as the ultrathin film is conductive.

**Figure 7:**
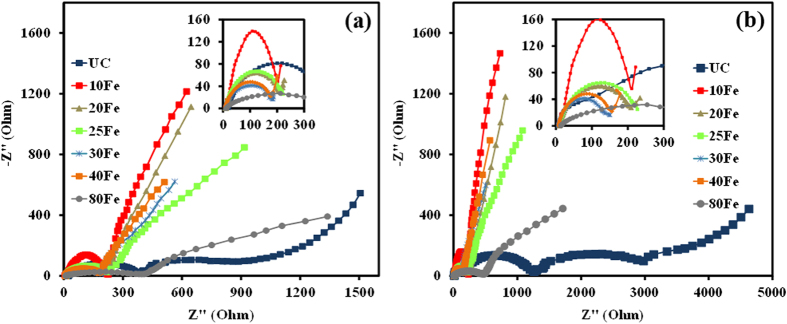
Electrochemical impedance spectra at 55 °C for uncoated LiMn_1.5_Ni_0.5_O_4_ particles and coated with various thicknesses of iron oxide after (**a**) 1^st^ cycle and (**b**) 1,000^th^ charge-discharge cycles. Inset images show the high frequency regions (1M Hz–100 Hz) of the impedance spectra.

EIS study was also performed at high temperature (55 °C), as shown in Fig. 7. The UC sample experienced much more increment in charge transfer resistance than the iron oxide ALD coated samples except for the 80Fe sample. The higher impedance of the UC sample at an elevated temperature has been attributed to the degradation reactions between the cathode and the electrolyte^3^. As discussed above, the 80Fe sample experienced large stresses coupled with high mass transfer resistance due to the relatively thick coating. That could be due to high charge transfer resistance from the distorted lattice structure. Comparing the impedance parameters of the 30Fe and 40Fe cells with the UC cell, it is clear that the UC cell was experiencing slower kinetics after cycling. The 30Fe cell showed the best results among all the other cells tested. With increase in charge-discharge cycling, the charge-transfer and the film resistance increased, and the difference between the UC cell and the coated cells grew significantly. For example, after 1,000 charge-discharge cycles, the combined film and charge transfer resistance of the 30Fe was 173.9 Ω, while it was 300.5 Ω for the UC cell, which was greatly increased from the value of the fresh cell. The resistance values explain that the kinetics of the surface film developed on the electrodes^31^,^32^. R_ohm_ values for the UC sample and the other samples are not the same. The change could be due to the structure modification of LMNO by iron doping and iron oxide coating. The 30Fe sample performs the best as compared to the other samples. This is because of the lowest charge transfer and film resistance of the 30Fe sample. For the 20Fe sample, the film was just not thick enough to provide good protection as compared to the optimal coating of 30Fe. Lower charge tranfer and film resistance could also mean that more Li^+^ ions are available at the 30Fe cathode surface, thereby compensating for increased diffusion resistance. The lower film resistance is due to the conductive iron oxide film coating. The trend of the charge transfer and the film impedance values confirm that the 30Fe sample has the optimal ultrathin coating of iron oxide.

**Figure 8:**
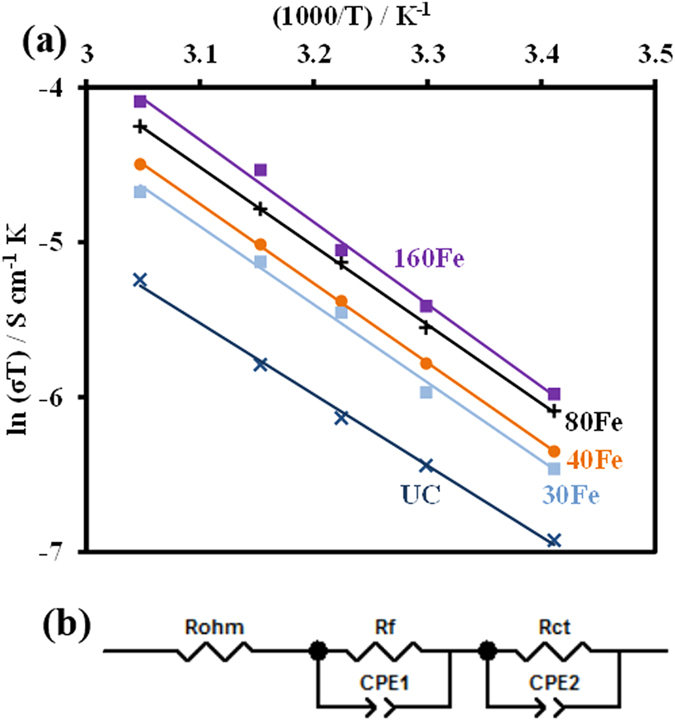
(**a**) Arrhenius plot of the UC and 30Fe, 40Fe, 80Fe, and 160Fe coated LiMn_1.5_Ni_0.5_O_4_ particles for the effects of temperature on conductivity, and (**b**) equivalent circuit for impedance spectra.

Pellets of only the UC, 30Fe, 40Fe, 80Fe, and 160Fe particles were prepared for conductivity measurements. The ac complex plane impedance analysis was used for the experiment^33^,^34^ and the same impedance analyzer was used to obtain the impedance curves. This experimental procedure was similar to the conductivity experiments carried out in our recent study^17^. The fitting of those curves were also perfomed and equivalent circuits were obtained. The equivalent circuit used to fit the impedance curves is shown in Fig. 8b. The equivalent circuit does not contain the warburg element as there is no conduction or movement of ions during this experiment. The obtained film resistance (R_f_) and charge transfer resistance (R_ct_) are combined in series to obtain an equivalent resistance value, which is used for conductivity calculations. For measuring the resistivity, the pellet thickness and diameter were used to calculate the area. This procedure helps us to calculate only the mixed ionic and electronic conductivites. Our work on the sepearation of these coductivities including the detailed methods and analysis will be illustrated in a future publication. Comparing the results among the uncoated and the coated samples (Fig. 8), which were prepared using the same procedure and material compositions, it is certain to conclude that iron oxide coating can improve the conductivity of the LMNO particles. The 160Fe sample shows the best conductivity compared to any other samples, which could also be true due to the presence of highly conductive Fe_3_O_4_ (or γ-Fe_2_O_3_). This is in contrast to our previous work^17^, where the highest conductivity was achieved with an optimum CeO_2_ thickness of 3 nm. In this case, the iron oxide ALD film growth rate was very low (the thickness of 160 cycles of iron oxide ALD is only 3 nm) and, hence it is thin enough to provide better conductivity for the coated samples with higher number of ALD cycles. The conductivity has been found to obey the Arrhenius equation^33^,^34^,


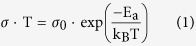


where, *σ*_0_ is the pre-exponential factor, 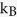 is the Boltzmann contant, T is the absolute temperature, and 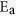 is the activation energy for Li ion movement. Figure 8 shows the direct co-relation between the mixed conductivity and the temperature (a linear Arrhenius plot). Since the testing temperatures were limited to 328 K, there was no phase or structural change observed during the measurements.

## Conclusions

In summary, we have successfully demonstrated that the cycle life and the capacity retention of LMNO can be significantly improved by the synergetic effect of ultrathin film coating of iron oxide combined with Fe ionic doping in the lattice structure LMNO particles. The ionic Fe penetration into the lattice structure of LMNO was verified by cross-sectional STEM-EDS of iron oxide coated samples and the ultra thin iron oxide films were directly observed by TEM. Mössbauer and XPS results confirmed the valance state of the iron for the ALD coated samples. It can be seen that the 30Fe sample has a high initial capacity of 143 mAh/g, which is about 25% higher than that of the UC sample at a 1C rate. It shows 93% capacity retention after 1,000 cycles at room temperature. More importantly, at elevated temperatures, the 30Fe sample performs the best as compared to the UC sample and other iron oxide coated samples. This work reports the first time the synergetic effect of doping and thin film coating on LMNO particles. ALD coating of iron oxide provided much better improvement in performance of LMNO than what could potentially be due to only doping effect. ALD has the potential to prepare these ultrathin electrochemically active films with optimal thickness and synergetic effect of conductive coating and element doping, providing the industry to design novel electrodes that are durable as well as functional at high temperature and fast cycling rates. Further in depth analysis of this unique technique could provide major breakthrough to solve the current shortcoming in the field of energy storage.

## Methods

### ALD coating

The ALD coating was carried out in a fluidized bed reactor, described elsewhere in detail^35^. There was a filter employed to contain the particles in the reactor, while allowing only gas to pass. Ferrocene (99% pure, Alfa Aesar) and oxygen (99.9%, Airgas) were used as precursors, and were delivered into the reactor in alternate doses at 450 °C. Ferrocene was delivered into the reactor using a heated bubbler and nitrogen was used as a carrier gas. Then N_2_ was used to purge the reactor to remove any unreacted ferrocene and by-products. After that, O_2_ was fed into the reactor, followed by another N_2_ purge. All lines were heated to 120 °C to avert any vapor deposition inside the lines.

### Materials characterization

The iron oxide films were verified using a FEI Tecnai F20 field emission gun high resolution TEM equipped with EDS system. ICP-AES was used to quantify the mass percent of iron on the particles. To check the Fe element distribution within the particles, about 80 nm thick thin section across the center of the particle was prepared by focused ion beam, using an FEI Q3D dual-beam system. The thin section was subsequently checked by a JEOL 2010F TEM in both TEM mode and scanning TEM mode at 200kV acceleration voltage. The crystal structure of the uncoated and coated particles was determined via powder XRD (Phillips Powder Diffractometer, CuKα radiation, λ = 1.5406 Å). The PXRD analysis was performed using a scan rate of 2°/min and a step size of 0.2°.

^57^Fe Mössbauer spectroscopy was performed on the 160Fe sample in transmission geometry using a constant acceleration spectrometer equipped with a ^57^Co (25 mCi) gamma source embedded in Rh matrix. The instrument was calibrated for velocity and isomer shifts with respect to α-Fe foil at room temperature. The resulting Mössbauer spectrum was analyzed using Lorentzian profile fitting by RECOIL software^36^.

### Coin cell assembly

An 80:10:10 wt.% mixture of LiMn_1.5_Ni_0.5_O_4_, carbon black (super P conductive, 99+%, Alfa Aesar) and polymer binder poly(vinylidene fluoride) (Alfa Aesar) was used to prepare cathodes. The slurry of the mixture was spread on the Al-foil, and then it was dry-heated at 120 °C. The cathode discs/working electrodes were made after the coated foil was punched. The reference/counter electrode was Li metal (99.9% trace metal basis, Sigma-Aldrich) and LiPF_6_ (1 mol/L in a mixed solvent of ethylene carbonate, dimethyl carbonate, and diethyl carbonate with a volume ratio of 1:1:1, MTI Corporation) was used as an electrolyte in all the cells prepared. The CR2032 cells fabrication was carried out in an Ar-filled glove box.

### Electrochemical analysis

The charge-discharge analysis was carried out using an 8-channel battery analyzer (Neware Corporation) for 3.5–5 V potential range at various C rates, and at different temperatures (room temperature and 55 °C). The electrochemical impedance spectroscopy measurements of the prepared cells were carried out using an IviumStat impedance analyzer. The EIS analysis was performed at 5 mV excitation signal and 0.001–1M Hz frequency range. Conductivity measurements were carried out using the same analyzer for cold pressed pellets of the samples. The pellets were coated with Ag (paste from Sigma Aldrich) on both sides to act as the blocking electrodes. These pellets were vacuum-dried at ~85 °C for 6 hr. The analysis was performed for a range of 1 Hz to 1 MHz and at 1 mV. The test temperature range was 20–55 °C. The spectra were analyzed using Zview software (Scribner Associates, Inc.). The conductivity tests were performed to compare the coated and uncoated samples and to examine the conductive nature of the coating with respect to the substrate only. Necessary steps were taken to make sure that all the cells and pellets were exposed to the same conditions for their respective batches of experiments.

## Additional Information

**How to cite this article**: Patel, R. L. *et al.* Employing Synergetic Effect of Doping and Thin Film Coating to Boost the Performance of Lithium-Ion Battery Cathode Particles. *Sci. Rep.* **6**, 25293; doi: 10.1038/srep25293 (2016).

## Supplementary Material

Supplementary Information

## Figures and Tables

**Table 1:**
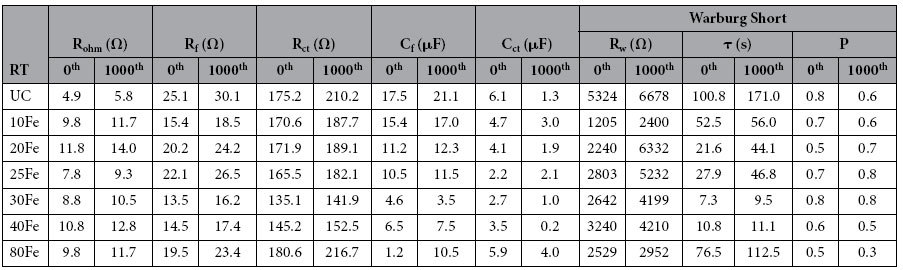
Impedance parameters using equivalent circuit models for electrodes made of UC, 10Fe, 20Fe, 25Fe, 30Fe, 40Fe, 80Fe coated LiMn_1.5_Ni_0.5_O_4_ particles at room temperature.

**Table 2:**
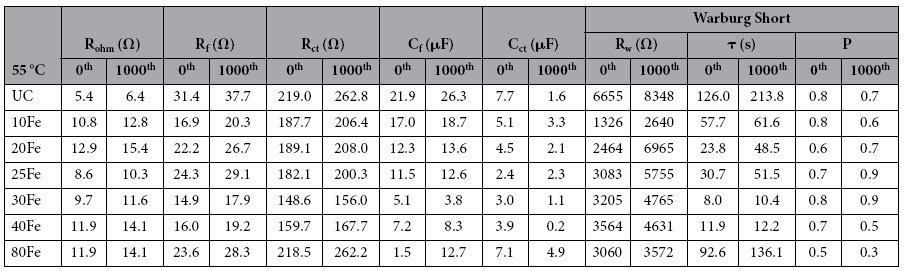
Impedance parameters using equivalent circuit models for electrodes made of UC, 10Fe, 20Fe, 25Fe, 30Fe, 40Fe, 80Fe coated LiMn_1.5_Ni_0.5_O_4_ particles 55 °C.
