# Supplementary material for: Employing Synergetic Effect of Doping and Thin Film Coating to Boost the Performance of Lithium-Ion Battery Cathode Particles
Source: Sci Rep. 2016 May 4;6:25293. doi: 10.1038/srep25293 (PMC4855153; doi:10.1038/srep25293)
Supplement: Supplementary Information [file srep25293-s1.pdf]

Supporting Information

Employing Synergetic Effect of Doping and Thin Film Coating to Boost the Performance of Lithium-Ion Battery Cathode Particles

Rajankumar L. Patel[1], Ying-Bing Jiang[2], Amitava Choudhury[3] & Xinhua Liang[1,*]

[1] Department of Chemical and Biochemical Engineering, Missouri University of Science and Technology, Rolla, Missouri 65409, United States
[2] TEM Laboratory, University of New Mexico, Albuquerque, New Mexico 87131, United States
[3] Department of Chemistry, Missouri University of Science and Technology, Rolla, Missouri 65409, United States

* Corresponding author, Email: liangxin@mst.edu

Structural Characterization

The selective area electron diffraction (SAED) patterns obtained, aligned along their main axis, from the UC and the 160Fe samples shown in Figures S2a and S2b, respectively, clearly demonstrated the differences in the structure for $LiMn_{1.5}Ni_{0.5}O_4$ (LMNO) before and after iron oxide ALD coating, which was also confirmed by unidentified peaks in XRD spectra of 160Fe (Figure 2). In comparison, pristine LMNO showed fewer lattice peaks in the spinal diffraction pattern in the (100) zone than the 160Fe. These extra peaks, for the case of 160Fe, correspond to the cubic phase ($P4_332$)[1].

X-ray photoelectron spectroscopy (XPS, Kratos Axis 165) was used to study the oxidation state of Fe by employing Al K (α) excitation, operated at 150 W and 15 kV. The peaks were corrected with C 1s at 284.6 eV. The values for the $Fe2p_{3/2}$ peak reported in the literature differs by 0.9 eV between two extreme values 710.6 eV [2] and 711.5 eV[3]. As shown in Figure S5, the 30Fe and 40Fe samples show a very sharp peak of Fe 2p at 711.5 eV, which is very close to the observed Fe (III) $2p_{3/2}$ in Fe_2O_3[4-7]. Also, these two samples show a small peak at 724 eV, which is very close to the observed peak of Fe (III) $2p_{1/2}$ in Fe_2O_3[4-8]. This indicates that the iron oxide deposited by ALD is Fe_3O_4 or γ-Fe_2O_3 as peaks for Fe (II) as well as Fe (III) oxidation state was observed. A faint confined peak at 710.1 eV for the 40Fe sample is representing Fe_3O_4. There is also a small satellite peak at ~717 eV in the 30Fe and 40Fe samples, which indicates the existence of Fe_2O_3, as reported by Gota et al[9]. Iron at an intermediate oxidation state in Fe_3O_4 with a mixture of Fe (II) and Fe (III) presents a BE value of 710.2 eV. The Fe^{3+} component of Fe $2p_{3/2}$ in γ-Fe_2O_3 is at 710.1 eV. The peak-shifts for 30Fe, 40Fe, and 80Fe samples are because that the main difference between the two sets of samples is the coordination of the Fe^{3+} cations. In the α-compounds, the crystal

structure is oriented in such a way that all of the cations are octahedrally coordinated. In the γ-compounds, on the other hand, three-quarters of the Fe^{3+} cations are octahedrally coordinated whereas the other quarter of the cations are tetrahedrally coordinated. This also explains the satellite peaks, as proven in literature[10], the XPS Fe 2p spectrum of 40Fe possesses smaller satellite intensity as compared with that of 30Fe due to the larger Fe 3d to O 2p hybridization in 40Fe, which has higher amount of γ-Fe_2O_3. The formation of a conformal iron oxide ALD layer on the surface can act as an artificial solid permeable interface (SPI) layer and helps prevent electrolyte decomposition at higher voltage[6]. The XRD and SAED results indicate that Fe in some oxidation state has penetrated into the lattice structure of LMNO[11,12].

References

1 Hai, B., Shukla, A. K., Duncan, H. & Chen, G. The effect of particle surface facets on the kinetic properties of $LiMn_{1.5}Ni_{0.5}O_4$ cathode materials. *J. Mater. Chem. A* **1**, 759-769, (2013).

2 Hawn, D. D. & DeKoven, B. M. Deconvolution as a correction for photoelectron inelastic energy losses in the core level XPS spectra of iron oxides. *Surf. Interface Anal.* **10**, 63-74 (1987).

3 Konno, H., Sasaki, K., Tsunekawa, M., Takamori, T. & Furuichi, R. X-ray photoelectron spectroscopic analysis of surface products on pyrite formed by bacterial leaching. *Bunseki Kagaku* **40**, 609-616 (1991).

4 Yamashita, T. & Hayes, P. Analysis of XPS spectra of Fe^{2+} and Fe^{3+} ions in oxide materials. *Appl. Surf. Sci.* **254**, 2441-2449 (2008).

5 Wu, C., Zhang, H., Wu, Y.-X., Zhuang, Q.-C., Tian, L.-L. & Zhang, X.-T. Synthesis and characterization of $Fe@Fe_2O_3$ core-shell nanoparticles/graphene anode material for lithium-ion batteries. *Electrochim. Acta* **134**, 18-27 (2014).

6 Philippe, B., Valvo, M., Lindgren, F., Rensmo, H. & Edström, K. Investigation of the electrode/electrolyte interface of Fe_2O_3 composite electrodes: Li vs Na batteries. *Chem. Mater.* **26**, 5028-5041, (2014).

7 Hu, T., Xie, M., Zhong, J., Sun, H.-T., Sun, X., Scott, S., George, S.M., Liu, C.-S. & Lian, J. Porous Fe_2O_3 nanorods anchored on nitrogen-doped graphenes and ultrathin Al_2O_3 coating by atomic layer deposition for long-lived lithium ion battery anode. *Carbon* **76**, 141-147 (2014).

8 Zhu, X., Song, X., Ma, X. & Ning, G. Enhanced electrode performance of Fe_2O_3 nanoparticle-decorated nanomesh graphene as anodes for lithium-ion batteries. *ACS Appl. Mater. Interfaces* **6**, 7189-7197 (2014).

9 Gota, S., Moussy, J. B., Henriot, M., Guittet, M. J. & Gautier-Soyer, M. Atomic-oxygen-assisted MBE growth of Fe_3O_4 (1 1 1) on α-Al_2O_3 (0 0 0 1). *Surf. Sci.* **482–485,** 809-816 (2001).

10 Fujii, T., de Groot, F.M.F., Sawatzky, G.A., Voogt, F.C., Hibma, T. & Okada, K. In-situ XPS analysis of various iron oxide films grown by NO_2-assisted molecular-beam epitaxy. *Phys. Rev. B* **59**, 3195-3202 (1999).

11 Liu, J. & Manthiram, A. Understanding the improved electrochemical performances of Fe-substituted 5 V spinel cathode $LiMn_{1.5}Ni_{0.5}O_4$. *J. Phys. Chem. C* **113**, 15073-15079 (2009).

12 Kim, J. H., Myung, S. T., Yoon, C. S., Kang, S. G. & Sun, Y. K. Comparative study of $LiNi_{0.5}Mn_{1.5}O_{4-\delta}$ and $LiNi_{0.5}Mn_{1.5}O_4$ cathodes having two crystallographic structures: $Fd\bar{3}m$ and $P4_332$. *Chem. Mater.* **16**, 906-914 (2004).

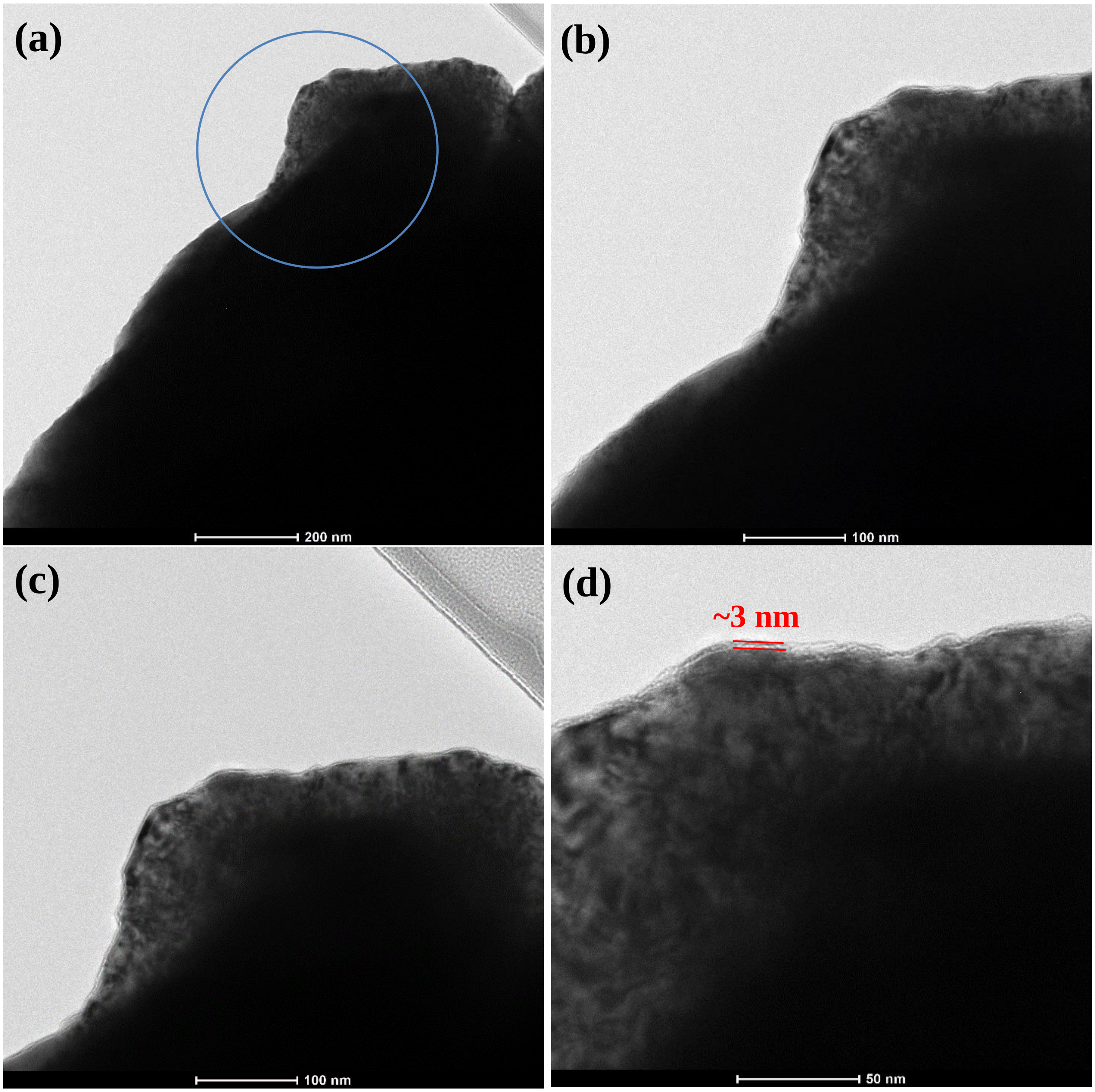

Figure S1. TEM images of 160 cycles of iron oxide ALD coated $LiMn_{1.5}Ni_{0.5}O_4$ particles. Conformal iron oxide film coverage shown in (a), (b), (c), and (d) at different magnification levels.

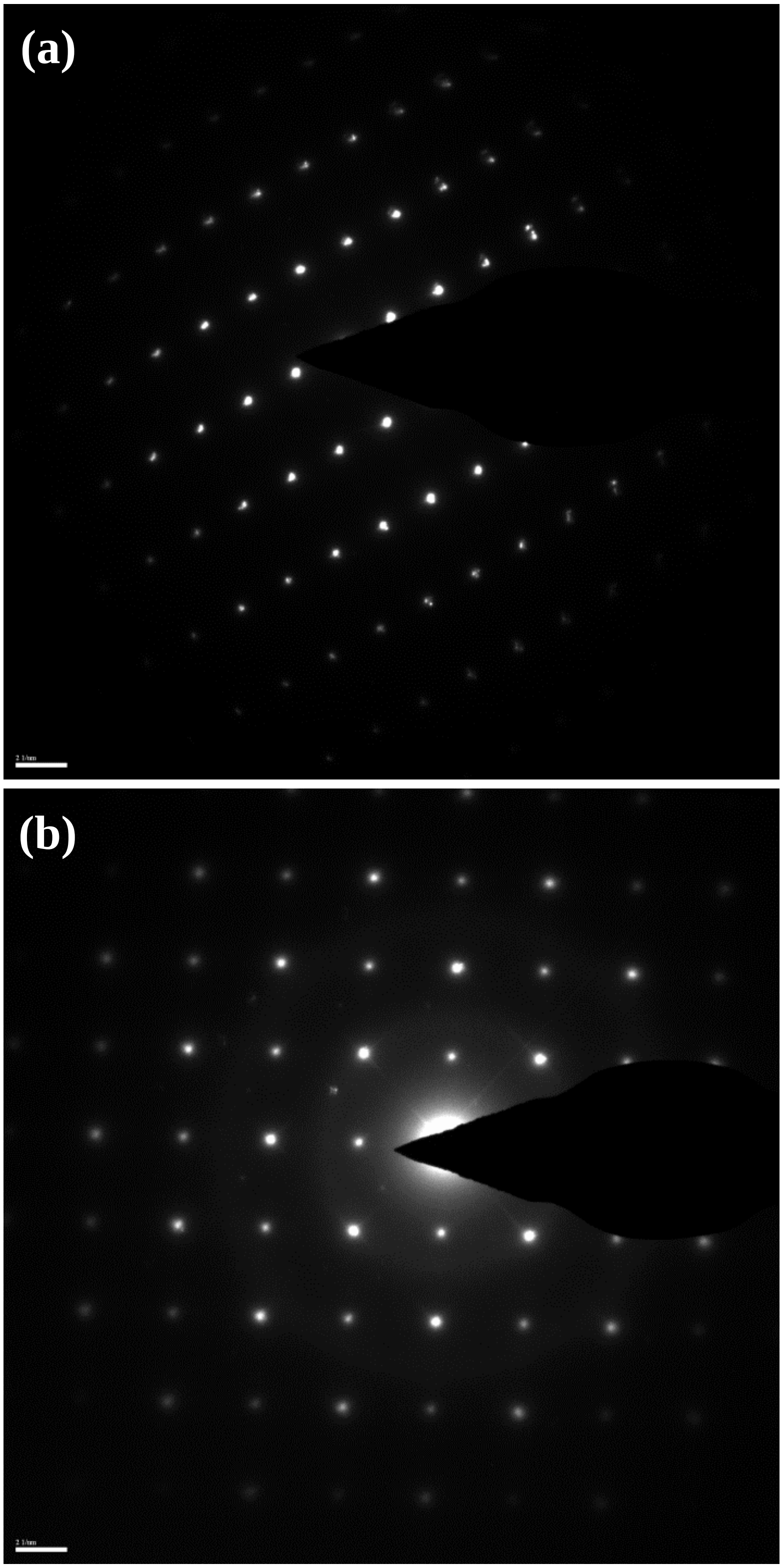

Figure S2. SAED patterns from TEM images of (a) uncoated, and (b) 160 cycles of iron oxide ALD coated $LiMn_{1.5}Ni_{0.5}O_4$ particles.

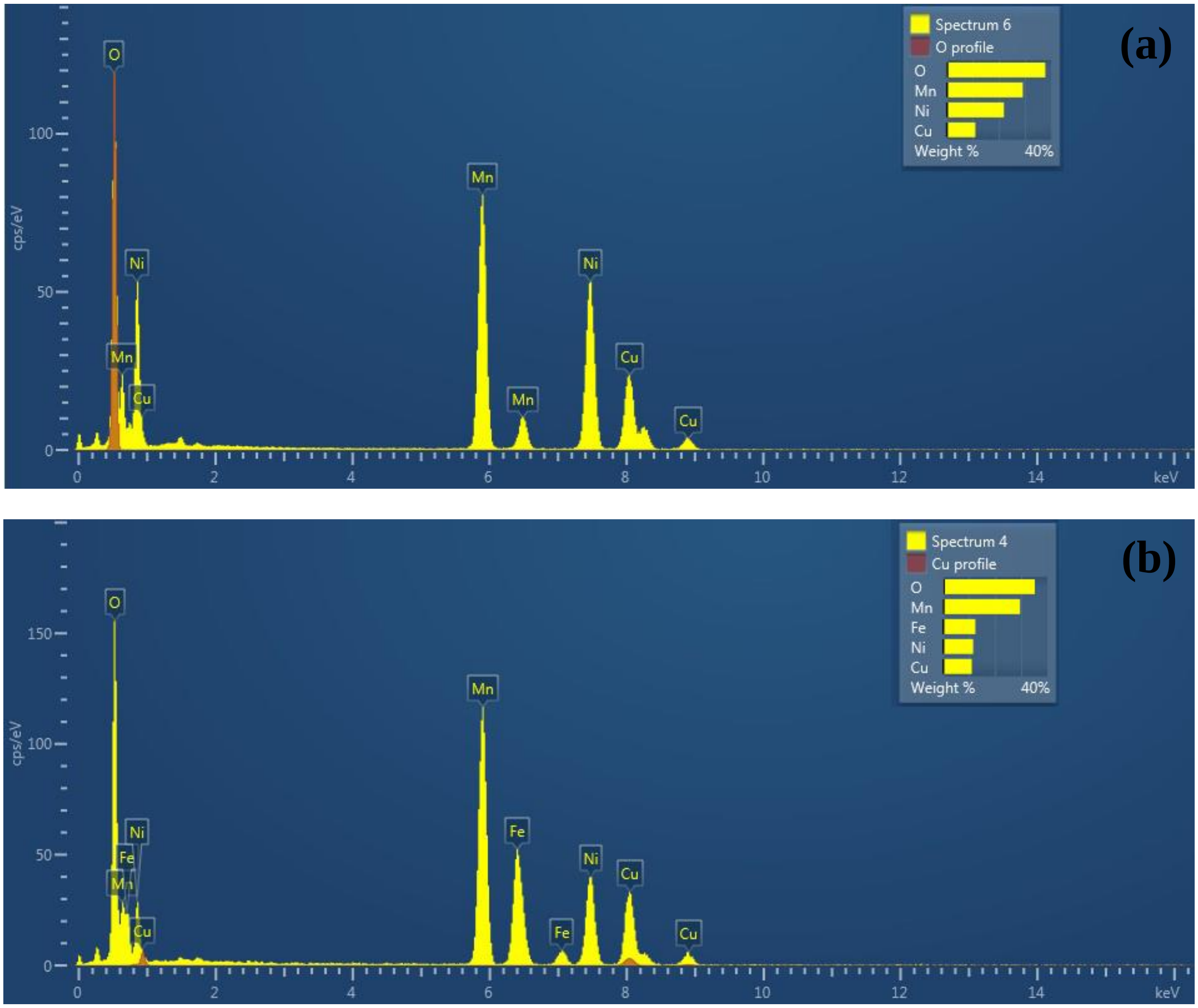

Figure S3. EDS spectra of (a) uncoated, and (b) 160 cycles of iron oxide ALD coated $LiMn_{1.5}Ni_{0.5}O_4$ particles.

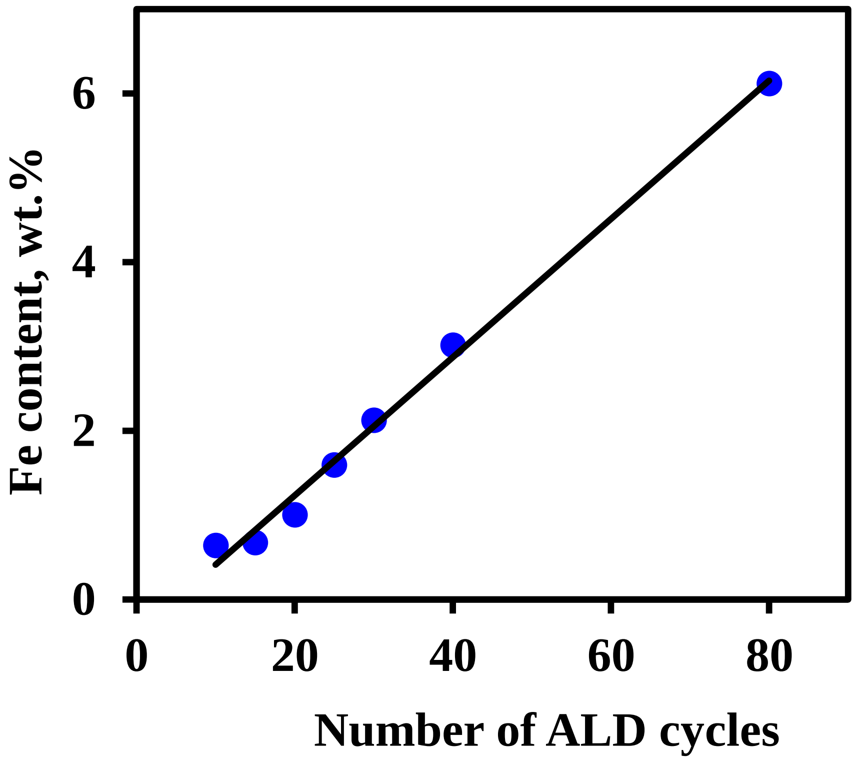

Figure S4. Iron contents on $LiMn_{1.5}Ni_{0.5}O_4$ particles versus the number of ALD coating cycles.

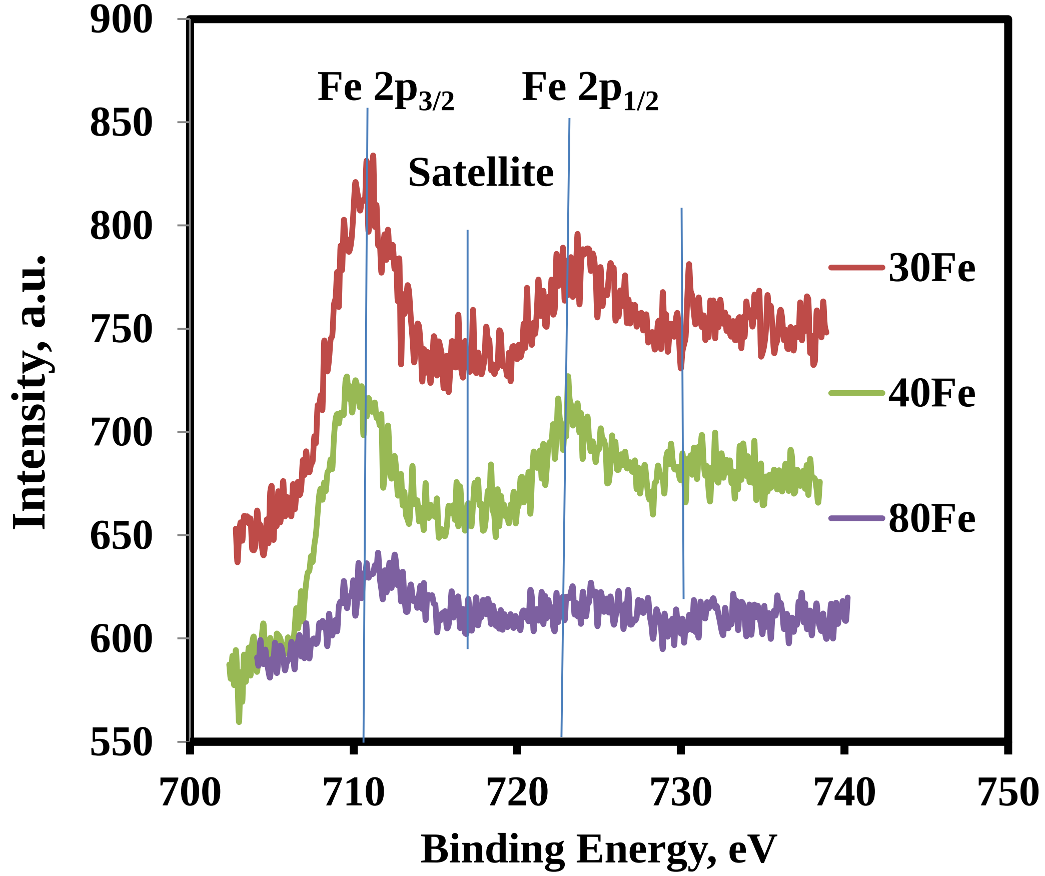

Figure S5. Fe 2p XPS spectra of 30, 40, and 80 cycles of iron oxide ALD coated $LiMn_{1.5}Ni_{0.5}O_4$ samples.

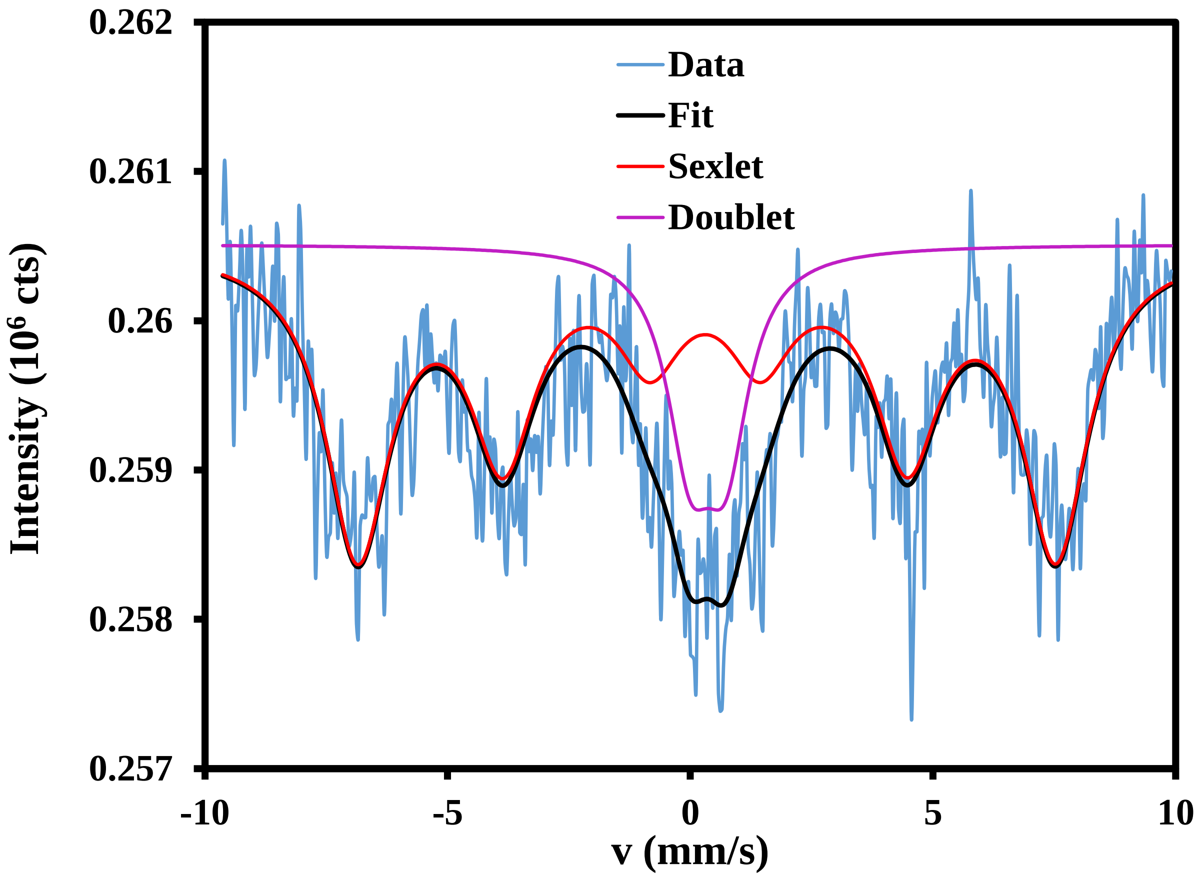

Figure S6. Mössbauer spectrum of the 160 cycles of iron oxide ALD coated $LiMn_{1.5}Ni_{0.5}O_4$ samples showing a sextet and a doublet site at the center.
